# Targeting KRAS mutations in pancreatic cancer: opportunities for future strategies

**DOI:** 10.3389/fmed.2024.1369136

**Published:** 2024-03-21

**Authors:** Anna Linehan, Mary O’Reilly, Ray McDermott, Grainne M. O’Kane

**Affiliations:** ^1^Department of Medical Oncology, St Vincent’s University Hospital, Dublin, Ireland; ^2^Department of Medical Oncology, St James’s Hospital, Dublin, Ireland; ^3^Princess Margaret Cancer Centre, Toronto, ON, Canada

**Keywords:** KRAS, mutation, pancreatic cancer, tumor microenvironment, SHP2, metabolic impact

## Abstract

Targeting the RAS pathway remains the holy grail of precision oncology. In the case of pancreatic ductal adenocarcinomas (PDAC), 90–92% harbor mutations in the oncogene KRAS, triggering canonical MAPK signaling. The smooth structure of the altered KRAS protein without a binding pocket and its affinity for GTP have, in the past, hampered drug development. The emergence of KRAS^G12C^ covalent inhibitors has provided renewed enthusiasm for targeting KRAS. The numerous pathways implicated in RAS activation do, however, lead to the development of early resistance. In addition, the dense stromal niche and immunosuppressive microenvironment dictated by oncogenic KRAS can influence treatment responses, highlighting the need for a combination-based approach. Given that mutations in KRAS occur early in PDAC tumorigenesis, an understanding of its pleiotropic effects is key to progress in this disease. Herein, we review current perspectives on targeting KRAS with a focus on PDAC.

## Introduction

**Figure 1:**
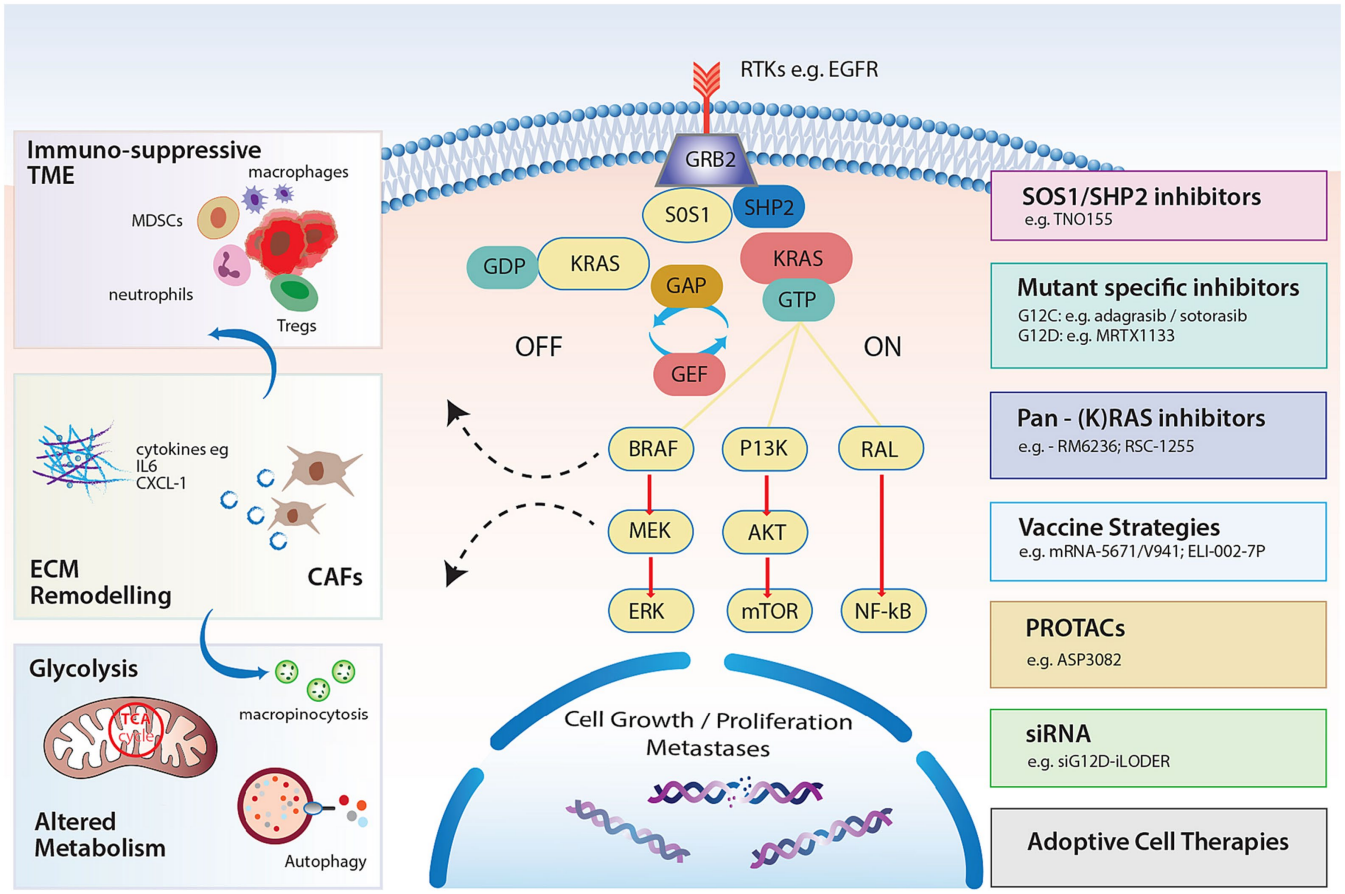
KRAS signaling pathways and impact on the TME, stroma and metabolism with strategies to target RAS.

The Kirsten rat sarcoma viral oncogene (KRAS) encodes the protein KRAS and represents the most altered protein across solid tumors underscoring the need for successful targeting (1). KRAS which is anchored as a membrane-bound protein in all human cells, acts as a central node for multiple signaling pathways important in normal cellular function (2). KRAS cycles between its inactive and active forms, facilitated by the exchange of KRAS-bound guanine diphosphate (GDP) and guanine triphosphate (GTP). Guanine exchange factors (GEFs) and GTPase activating proteins (GAPs) orchestrate this exchange and are therefore critical in maintaining KRAS activity (Figure 1). This well-controlled cycle was long considered ‘undruggable’ due to two main factors: the high affinity of KRAS for GTP and the absence of appropriate binding pockets suitable for the development of small molecule inhibitors (3, 4). The last 10 years have heralded a new era in personalized medicine with the discovery of the covalent KRAS^G12C^ inhibitors and a plethora of new investigational KRAS targeting agents are now entering the clinic. Innate and acquired resistance mechanisms to KRAS inhibitors have already been documented and our understanding of the pleiotropic effects of oncogenic KRAS on the tumor microenvironment has evolved. This has opened up new avenues in the treatment of KRAS mutated tumors with combination-based approaches in addition to emerging vaccines and adoptive cell therapy (ACT) strategies. Although oncology has seen the evolution of agnostic biomarkers to facilitate precise approaches, it remains to be seen whether mutations in the KRAS gene will represent universal biomarkers or whether inhibitor effects will be cell lineage dependent. In particular, pancreatic cancer has been a global challenge from a therapeutic perspective. Herein, we review approaches to KRAS targeting in pancreatic cancer to date and suggest some challenges for the future.

## KRAS signaling

KRAS is located on the short arm of chromosome 12 and is a member of the RAS oncogene family along with 2 other isoforms, neuroblastoma rat sarcoma viral oncogene (NRAS) and Harvey rat sarcoma viral oncogene (HRAS) (chromosomes 1 and 11 respectively) (5). KRAS is composed of 2 major domains, a catalytic domain called the G domain, and a hypervariable region at the C-terminal (6). The G domain consists of 3 regions: switch I, switch II and the P loop. Notably, as KRAS cycles between active and inactive states, the switch I and II pockets alter their conformation, providing a therapeutic vulnerability.

In the resting state, KRAS binds to GDP, which does not activate downstream signaling and is therefore considered “OFF.” GEFs limit KRAS-GDP affinity and act to replace GDP for GTP when activated by extracellular signals in the form of growth factors, e.g., epidermal growth factor (EGF), cytokines, and other molecules (Figure 1). Son of sevenless 1 and 2 (SOS1/SOS2), growth factor receptor-bound protein (GRB2) and RAS protein-specific guanine nucleotide-releasing factor 2 (RASGRF2) are common GEFs that enable the exchange (7). Src homology-2 domain containing protein tyrosine phosphatase (SHP2), which is considered a scaffolding protein, facilitates the GRB2/SOS1 complex at the cell membrane through direct binding to GRB2, promoting KRAS activation (8).

When KRAS is bound to GTP, it is considered “ON,” activating downstream signaling cascades. To convert back to the “OFF” state, KRAS-GTP must undergo hydrolysis. Intrinsic hydrolysis of GTPases is relatively ineffective and requires the assistance of GTPase-activating proteins (GAPs) such as neurofibrin-1 (NF1) or p120-RasGAP protein (encoded by RASA1) (9).

When GTP-bound, subsequent interaction with RAS-binding domains (RBDs) of effector proteins permits downstream signaling (10). The three major RAS effectors include rapidly accelerated fibrosarcoma (RAF) proteins, ral guanine nucleotide dissociation stimulator (RALGDS) and the phosphoinositide 3-kinases (PI3Ks) (Figure 1). Perhaps the most notable interaction is the activation of the mitogen activated protein kinase pathway (MAPK) RAF–MEK–ERK pathway. Activated KRAS ignites phosphorylation of RAF and subsequently extracellular signal regulated kinase (ERK) 1 and 2 following RAF dimerization (11). Phosphorylation of ERK results in ERK translocation to the nucleus where further transcriptional factors are phosphorylated and cell cycle progression through G0/G1 mitogenic signals ensues (12). Notably, the degree of activation of RAF kinases is likely variable which may influence prognostic outcomes of different mutated alleles (13).

Cellular growth and proliferation also result from signaling through the PI3K/protein kinase B(AKT) pathway. Activated PI3K phosphorylates phosphatidylinositol(PIP)2 to PIP3 promoting AKT phosphorylation which in turn stimulates many downstream pathways and regulates cell proliferation, cell death and metabolic processes, including cellular responses to insulin (14). Interactions with mTOR target proteins via feedback phosphorylation promote cell proliferation but also activate Bcl-XL/Bcl-2 to facilitate apoptosis (15, 16). The third major effector of RAS is the Ral guanine nucleotide exchange factor pathway. Ral proteins (RalA and RalB) are GTPases also cycling between GDP and GTP bound states. This effector of RAS complements RAS/RAF and RAS/PI3K signaling and is known to stimulate the Jun-N terminal kinase pathway promoting cell migration and proliferation (17).

### Altered KRAS signaling

#### Mutations

Oncogenic mutations in KRAS typically occur at hotspots in the protein with 95% occurring in codons 12, 13, and 61 (18). In pancreatic cancer the most common mutations occur on codon 12 with a single amino acid missense mutation when a glycine (G) is replaced by aspartate (G12D, 40%), valine (G12V, 29%), arginine (G12R, 15%), and cystine (G12C ~1%) (19). Less common mutations include G13 and Q61. KRAS^Q61H^ is found in about 5% of pancreatic cancer and is selectively associated with increased survival (20, 21). In contrast, KRAS^G12D^ has been associated with worse outcomes (22).

Mutations in KRAS increase KRAS-GTP affinity and the conformational changes inhibit GAP-mediated hydrolysis while also ensuring resistance to intrinsic GTPase activity (23, 24). In fact, it is thought that mutated KRAS may be more reliant on intrinsic rather than GAP-mediated hydrolysis to achieve its off state. Importantly, there are distinct biological differences in mutated KRAS forms. Mutations in codons 12 and 61 are insensitive to NF-1 mediated hydrolysis (25) and KRAS^G12C^ mutations demonstrate intrinsic GTPase activity similar to wild-type KRAS allowing for cycling between GDP and GTP bound states (23). In evaluating G12 mutants, higher intrinsic GTPase activity has been shown in G12D and G12C compared to G12R and G12V (23).

The etiology of some KRAS mutant cancers are distinct, e.g., G12C is the most common KRAS mutation in smoking-associated non-small cell lung cancer (NSCLC) (26). However, associations with epidemiologic risk factors have not been documented in PDAC.

#### KRAS amplification

Abnormal KRAS signaling in cancer can also be identified through high-level amplification of the KRAS gene. This is the fourth most common KRAS alteration that occurs and is evident in esophageal, stomach and serous ovarian cancer, generally representing an aggressive subtype (27, 28). Moreover, KRAS amplification permits adaptive resistance to MAPK inhibition and highlights the need for combination approaches (28). In pancreatic cancer, the dosage of mutant KRAS underpins particularly aggressive phenotypes (29). Major imbalances in mutant versus wild-type KRAS reflect the basal-like subtype and are associated with an inferior outcome in both resected and advanced PDAC (29, 30). This phenotype of PDAC is more evident in metastases than primaries and polyploid tumors, suggesting that evolutionary components to PDAC progression dictate more aggressive subtypes.

#### Common co-mutations

In pancreatic cancer, KRAS mutations commonly occur alongside inactivating mutations in tumor suppressor genes such as TP53, CDKN2A, and SMAD4 (31). Sequential inactivation of these genes is important in the evolution of invasive disease (32). This inactivation contributes to the heterogeneous nature of KRAS mutated PDAC with the number of drivers influencing survival (22, 33). Co-mutations in STK11 and the PIK3/AKT/mTOR pathway further contribute to inferior outcomes (34).

## KRAS and progression to invasive PDAC

There are two predominant precursor lesions to invasive PDAC, namely pancreatic intraepithelial neoplasia (PanIN) (85–90%) and intraductal papillary mucinous neoplasms (IPMNs) (10–15%). KRAS mutations are evident even in low-grade PanIN highlighting its principal role early in oncogenesis but also underscoring the need for the acquisition of co-mutations for invasive disease to develop. Indeed a stepwise model for progression has long been accepted (35, 36), although alternate models propose large-scale chromothriptic events which permit rapid disease progression, bypassing traditional models (37). Increasing knowledge of the malignant potential of cystic lesions, which are readily apparent on imaging, has revealed recurrent genetic drivers in KRAS, GNAS and RNF43, with additional alterations seen in invasive disease (38–40). With the successive inactivation of tumor suppressors in PDAC, cell cycle progression increases, as measured by RNA sequencing or Ki-67 (41). Transcriptional subtypes from primaries and metastases document major classifiers as basal-like and classical, with hybrid phenotypes evident which suggests tumoral plasticity. Although the incidence of the basal-like subtype is more apparent in metastases and associates with major imbalances in mutant KRAS, it is not yet clear what drives the progression to this phenotype. The influence of the tumor microenvironment (TME) and epigenetic factors are likely to be important. A full understanding of the progression of precursor lesions to invasive disease will underpin where KRAS can be targeted and what combination strategies may be needed.

## Impact of KRAS signaling on the tumor microenvironment and stroma

PDAC harbors a unique tumoral niche characterized by a dense fibrotic stromal reaction and an immunosuppressive TME. Infiltration of immunosuppressive cells, encouraging immune evasion, is thought to occur early, highlighting the cell-extrinsic impacts of KRAS mutations (41, 42). In fact, it has been well documented in the genetically engineered KPC (*Kras ^LSL-G12D/+Trp53LSL-R172H/+^ Pdx-Cre*) and KC mouse (*Kras^LSL-G12D/+^ Pdx-Cre*) models, that KRAS mutations in PanIN lesions associate with inflammatory environments with overexpression of COX2 (42), and early infiltration of T regulatory cells (Tregs), Tumor associated macrophages (TAMs) and Myeloid derived suppressor cells (MDSCs). This indicates a shift toward early immune evasion (43) and KRAS mutations induce multiple cytokines to promote and maintain this tumor promoting environment in invasive disease. RAS mutations induce the secretion of IL-6 in different cell types including myeloid cells, resulting in JAK1 activation and phosphorylation of the STAT3 pathway, culminating in a pro-inflammatory TME and shifting homeostasis toward tumorigenesis (44–46). Additionally, granulocyte macrophage colony stimulating factor (GM-CSF) secretion in response to mutated KRAS, can occur early and promotes MDSC infiltration, restricting anti-tumor immunity (47, 48). Moreover, continued secretion by mesenchymal stem cells within the stroma encourages tumor invasion and metastasis formation (49, 50) (Figure 1). Pivotal studies have demonstrated the upregulation of chemokines including CXCL-8 (IL-8) (51) and CXCR2 (52, 53) in response to RAS signaling, together with transcription factors such as NF-κB, promoting inflammation (54). Immune escape is further reinforced through the upregulation of PD-L1 and the conversion of CD4+ cells to Tregs (55). The latter is enhanced following ERK activation and secretion of IL-10 and TGF-B1, illustrating the importance of downstream pathways in shaping the TME (55). Nevertheless, the presence of a distinct chemokine signature (CCL4, CCL5, CXCL9, CXCL-10) can associate with T cell infiltration (56), and represents a biomarker of response to immune checkpoint inhibitor combinations in certain PDAC genotypes (57). This highlights the potential importance of profiling the TME for combination approaches.

The characteristic desmoplasia of PDAC is inherently reliant on cancer-associated fibroblasts (CAFs) with varying CAF subtypes identified (58). It is now known that targeting stroma may accelerate PDAC progression. The origin of CAFs remains unclear with a minority produced from the pancreatic stellate cell (59). Immunosuppressive and immune-enhancing CAFs exist (60), however oncogenic KRAS promotes non-cell autonomous programming of CAFs to stimulate cytokines, which regulate MDSCs and other immune cells (61). Understanding the pleiotropic effects of KRAS mutations in molding the TME early in oncogenesis will be critical in trial design of combination approaches in PDAC. Notably, the unselected use of immune checkpoint inhibitors has been unsuccessful to date (62).

## Metabolic impact on KRAS mutations

Cancer cell metabolism is critical to survival and in PDAC, KRAS mutations shape a unique metabolic environment which drives aerobic glycolysis (63). The adaptive switch to produce glucose, glutamine and fatty acids facilitates a supply for growth and proliferation. Notably, RAS-dependent metabolic adaption is likely tumor-specific, owing to the prevalence of specific KRAS mutations (64, 65). Mutations in KRAS are known to upregulate the GLUT1 transporter together with induction of enzymatic activity to increase glycolysis (66). Glutaminolysis also increases in the presence of oncogenic RAS signaling, providing glutamate from glutamine, fuelling tricarboxylic acid cycle (TCA) and providing a pool of amino acids (67, 68). Nutrient scavenging pathways such as autophagy are known to be upregulated in PDAC (69), and continue to feed the TCA cycle sustaining growth (70). Targeting autophagy has been a huge therapeutic strategy in the last number of years with multiple trials ongoing with hydroxychloroquine. Studies have shown that targeting RAS effector pathways such as ERK signaling may increase reliance on autophagy, necessitating combination approaches (71). Macropinocytosis is an additional metabolic process where cells can engulf extracellular material and is thought essential for PDAC survival (72, 73). The transcription factor MYC is important in this pathway, and notably, recent work has established mechanistic differences in macropinocytosis according to the mutant allele. KRAS^G12R^ exhibits impaired activation of the effector p110alpha PI3K, exposing potential therapeutic vulnerabilities (64). The crosstalk between KRAS, the TME and metabolic pathways will be critical in advancing combination therapeutic strategies, especially given the recent disappointments in targeting metabolism in the AVENGER trial (74).

## Current KRAS targets

### Allele-specific KRAS inhibitors

#### Direct G12C inhibitors

To date, progress in the field has been dominated by KRAS^G12C^ inhibitors, however, KRAS^G12C^ only represents approximately 1% of PDAC KRAS mutations (75). The direct G12C inhibitors bind covalently to the mutant cysteine, occupying the switch II pocket, trapping KRAS^G12C^ in the inactive, GDP-bound or “OFF” state (76).

Sotorasib (AMG-510) was the first direct KRAS^G12C^ inhibitor to enter the clinical arena. It is an oral small-molecule inhibitor that selectively and irreversibly targets KRAS^G12C^ and has been FDA-approved for the treatment of advanced NSCLC, based on the phase II CodeBreaK-100 trial. Patients with KRAS^G12C^ mutated NSCLC (*n* = 126) were treated with sotorasib 960 mg OD and achieved an objective response rate (ORR) of 37.1% (95% CI, 28.6–46.1), with a median progression-free survival (PFS) of 6.8 months (95% CI, 5.1–8.2) (4). The response in KRAS^G12C^ mutated colorectal cancer is notably inferior, with an ORR of just 9.7% (95% CI 3.6–19.1) seen in this cohort of the CodeBreaK-100 trial (77). This improved to 30% (95% CI, 16.6–46.5) in the phase Ib CodeBreaK-101 trial with the addition of panitumumab, a monoclonal antibody targeting epidermal growth factor receptor (EGFR) (78). A further improvement was seen with the combination of sotorasib, panitumumab and FOLFIRI (5-fluorouracil, leucovorin and irinotecan) in which 58.1% (95% CI, 39.1–75.5) had an objective response (79). The activity of sotorasib in advanced PDAC harboring KRAS^G12C^ has been disappointing thus far. The phase I/II CodeBreaK-100 trials included 38 patients with advanced PDAC and demonstrated an ORR of 21% (95% CI, 10–37) with a disease control rate (DCR) of 84.2% (95% CI, 68.7–93.9). The median PFS was 4.0 months (95% CI 2.8–5.6) with a median overall survival (OS) of 6.9 months (95% CI 5.0–9.1) (80) (Table 1).

**Table 1:** Response to single agent KRAS G12C inhibition in NSCLC, colorectal cancer and pancreatic cancer.

	NSCLC			Colorectal			Pancreas		
	Sotorasib	Adagrasib	Divarasib	Sotorasib	Adagrasib	Divarasib	Sotorasib	Adagrasib	Divarasib
	Phase III *n* = 171 (81)	Phase II *n* = 112 (82)	Phase I *n* = 58 (83)	Phase I/II *n* = 62 (77)	Phase II *n* = 43 (84)	Phase I *n* = 39 (83)	Phase I/II *n* = 38 (80)	Phase II *n* = 21 (85)	Phase I *n* = 7 (83)
ORR (%) 95% CI	28.1 (21.5, 35.4)	42.9 (33.5, 52.6)	53.4 (39.9, 66.7)	9.7 (3.6, 19.9)	19 (8, 33)	35.9 (21.2, 52.8)	21 (10, 37)	33.3 (14.6, 57.0)	43 (NR)
DCR (%) 95% CI	82.5 (75.9, 87.8)	79.5 (70.8, 86.5)	90 NR	82.3 (70.5, 90.8)	86 NR	NR	84 (69, 94)	NR	100 (NR)
DOR (mos) 95% CI	8.6 (7.1, 18.0)	8.5 (6.2, 13.8)	14.0 (8.3, NE)	4.2 (2.9, 8.5)	4.3 (2.3, 8.3)	7.7 (5.7, NE)	5.7 (1.6, NE)	5.3 (2.8, 7.3)	NR
mPFS (mos) 95% CI	5.6 (4.3, 7.8)	6.5 (4.7, 8.4)	13.1 (8.8, NE)	4.0 (2.8, 4.2)	5.6 (4.1, 8.3)	6.9 (5.3, 9.1)	4.0 (2.8, 5.6)	5.4 (3.9, 8.2)	NR
mOS (mos) 95% CI	10.6 (8.9, 14.0)	12.6 (9.2, 19.2)	NR	10.6 (7.7, 15.6)	19.8 (12.5, 23.0)	NR	6.9 (5.0, 9.1)	8.0 (5.2, 11.8)	NR

Adagrasib (MRTX849), another direct KRAS^G12C^ inhibitor with a longer half-life than sotorasib at 23 h, is dosed at 600 mg BD based on dose escalation in the phase I KRYSTAL-1 trial (86). The twice-daily dosing may have contributed to the improved response rates in NSCLC and colorectal cancer compared with sotorasib, at 42.9% (95% CI, 33.5–52.6) and 19% (8/43) respectively (82, 84). Again, the addition of an EGFR inhibitor cetuximab, improved the ORR in the colorectal cohort to 46% (95% CI 8–33) and demonstrated an increase in duration of response from 4.3 months (95% CI 2.3–8.3) to 7.6 months (95% CI 5.7-not estimable) (84). There were also improved responses in the PDAC cohort of the phase I/II KRYSTAL-1 trial (*n* = 21) with an ORR of 33.3% (95% CI 14.6–57.0) and a DCR of 81% (17/21). The median PFS was 5.4 months (95% CI, 3.9–8.2) and OS 8.0 months (95% CI 5.2–11.8) (85).

Divarasib (GDC-6036), a KRAS^G12C^ inhibitor which has increased potency and selectivity relative to sotorasib and adagrasib, has demonstrated superior response rates in a phase I trial of 137 patients with advanced KRAS^G12C^ mutated solid tumors (83). Treatment with divarasib resulted in an ORR of 53.4% (95% CI, 39.9–66.7) in NSCLC and 29.1% (95% CI 17.6–42.9) in colorectal cancer (Table 1). There were 7 patients with PDAC included in the trial, and of these, 3 (43%) experienced an objective response and 4 (57%) recorded stable disease (83).

Safety and efficacy data of multiple other compounds targeting KRAS^G12C^ has been presented in the past 12 months. This includes MK-1084, Garsorasib, JDQ443 (target GDP-bound KRAS) and IBI351 (targets GDP/GTP exchange). These have not yet reported data on PDAC cohorts. The LOXO-RAS-20001 trial of LY3537982 (targets GDP-bound KRAS) in KRAS^G12C^ mutated advanced solid tumors included 8 patients with PDAC and reported an ORR of 38% (87). RMC-6291 is a tricomplex inhibitor which acts similarly to the immunosuppressant cyclosporin by binding to cyclophilin A, targeting the GTP-bound state. It has shown promise in NSCLC including a partial response in 50% who had been previously treated with a KRAS inhibitor (88). These compounds may overcome resistance to GDP-bound inhibitors which can acquire secondary resistance mutations (89). There are numerous clinical trials of KRAS^G12C^ inhibitor agents ongoing (Table 2).

**Table 2:** Ongoing clinical trials of KRAS directed therapies in PDAC.

Clinical trial identifier	Agent	Phase	Mutation	Combination agent/comparator	Status
**KRAS^G12C^** **Inhibitors**			**KRAS** ^**G12C**^		
NCT05263986	Adagrasib	I	AST		Active, not recruiting
NCT05634525	Adagrasib	I	PDAC		Recruiting
NCT05178888-KRYSTAL-16	Adagrasib	I	AST	w/palbociclib	Active, not recruiting
NCT05848843	Adagrasib	I	GI/NSCLC	w/durvalumab	Not yet recruiting
NCT06039384	Adagrasib	I	AST	w/INCB099280	Recruiting
NCT06117371	BEBT-607	I	AST		Recruiting
NCT04973163	BI-1823911	I	AST	w/BI 1701963, midazolam	Active, not recruiting
NCT05315180	BPI-421286	I	AST		Recruiting
NCT05410145	D3S-001	I	AST		Recruiting
NCT04449874	Divarasib	I	AST	w/atezolizumab, cetuximab, bevacizumab, erlotinib, GDC-1971, inavolisib	Recruiting
NCT05768321	GEC255	I	AST		Recruiting
NCT05485974	HBI-2438	I	AST		Recruiting
NCT04956640-LOXO-RAS-20001	Ly3537982	I	AST	w/abemeciclib, erlotinib, pembrolizumab, temuterkib, LY3295668, cetuximab, TNO155	Recruiting
NCT05067283	MK-1084	I	AST	w/pembrolizumab	Recruiting
NCT05462717	RMC-6291	I	AST		Recruiting
NCT06006793	SY-5933	I	AST		Recruiting
NCT06130254	Adagrasib	Ib	AST	w/olaparib	Not yet recruiting
NCT06128551	RMC-6291	Ib	AST	w/RMC-6236	Not yet recruiting
NCT03785249-KRYSTAL-1	Adagrasib	I/II	AST	w/cetuximab, pembrolizumab, afatinib	Recruiting
NCT04585035	Garsorasib	I/II	AST		Recruiting
NCT05379946	Garsorasib	I/II	AST	w/IN10018	Enrolling by invitation
NCT05005234	IBI351	I/II	AST		Recruiting
NCT05367778	HS-10370	I/II	AST		Recruiting
NCT05009329	Glecirasib	I/II	AST		Recruiting
NCT05002270	Glecirasib	I/II	AST	w/cetuximab	Recruiting
NCT04699188-KontRASt-01	JDQ443	I/II	AST	w/TNO155, tislelizumab	Recruiting
NCT05358249-KontRASt-03	JDQ443	I/II	AST	w/trametinib, ribociclib, cetuximab	Recruiting
NCT03600883-CodeBreaK100	Sotorasib	I/II	AST	w/anti PD-1/L1	Active, not recruiting
NCT04185883-CodeBreaK101	Sotorasib	I/II	AST	w/AMG404, trametinib, RMC-4630, afatinib, pembrolizumab, panitumumab, atezolizumab, everolimus, palbociclib, bevacizumab, TNO155, FOLFIRI, carboplatin, pemetrexed, docetaxel	Recruiting
NCT04892017	Sotorasib	I/II	AST	w/DCC-3116	Recruiting
NCT05173805	YL-15293	I/II	AST		Enrolling by invitation
NCT06008288	Glecirasib	II	PDAC		Not yet recruiting
NCT05993455	Sotorasib	II	AST	w/panitumumab	Active, not recruiting
NCT05638295-ComboMATCH	Sotorasib	II	AST	w/panitumumab	Not yet recruiting
**KRAS**^**G12D**^ **Inhibitors**			**KRAS** ^**G12D**^		
NCT05533463	HRS-4642	I	AST		Recruiting
NCT06040541	RMC-9805	I/Ib	AST		Recruiting
NCT05737706	MRTX1133	I/II	AST		Recruiting
**KRAS**^Multi^ **Inhibitors**			**KRAS** ^**G12**^		
NCT05379985	RMC-6236	I	AST		Recruiting
NCT04678648	RSC-1255	I	AST		Recruiting
			**KRAS** ^**ANY**^		
NCT06078800	YL-17231	II	AST		Recruiting
NCT06096974	YL-17231	I	AST		Not yet recruiting
**SOS1 Inhibitors**			**KRAS** ^**ANY**^		
NCT04111458	BI-1701963	I	AST	w/trametinib	Active, not recruiting
NCT04973163	BI-1701963	I	AST	w/BI-1823911	Active, not recruiting
NCT05578092	MRTX0902	I/II	AST	w/adagrasib	Recruiting
**SHP2 Inhibitors**			**KRAS** ^**G12C**^		
NCT05480865-ARGONAUT	BBP-398	I	AST	w/sotorasib	Recruiting
NCT05010694	GH35	I	AST		Recruiting
NCT05163028	HBI-2376	I	AST		Recruiting
NCT04916236-SHERPA	RMC-4630	I	CRC/NSCLC/PDAC	w/LY3214996	Recruiting
NCT06024174	BMS-986466	I/II	AST	w/adagrasib, cetuximab	Recruiting
NCT05288205	JAB-3312	I/II	AST	w/glecirasib	Recruiting
NCT04418661	RMC-4630	I/II	AST	w/pembrolizumab. Adagrasib	Active, not recruiting
NCT04330664-KRYSTAL-2	TNO155	I/II	AST	w/adagrasib	Active, not recruiting
			**MAPK alterations**		
NCT05853367	MK-0472	I	AST	w/pembrolizumab	Recruiting
NCT03634982	RMC-4630	I	AST		Active, not recruiting
			**No mutation req.**		
NCT04800822	ARRY-558		AST	w/lorlatinib, binimetinib, encorafenib, cetuximab	Active, not recruiting
NCT04528836	BBP-398	I	AST		Recruiting
NCT05369312	BBP-442096	I	AST		Not yet recruiting
NCT05354843	ET0038	I	AST		Recruiting
NCT04670679-FLAGSHP-1	ERAS-601	I	AST	w/cetuximab	Active, not recruiting
NCT05378178	HS-10381	I	AST		Recruiting
NCT04045496	JAB-3312	I	AST		Recruiting
NCT05505877	BR-790	I/II	AST	w/tislelizumab	Recruiting
NCT04866134	ERAS-601	I/II	AST	w/ERAS-007	Active, not recruiting
NCT03565003	JAB-3068	I	AST		Recruiting
NCT04720976	JAB-3312	I/II	AST	w/binimetinib, pembrolizumab, sotorasib, osimertinib	Recruiting
**KRAS Degraders**			**KRAS** ^**G12D**^		
NCT05382559	ASP3082	I	AST		Recruiting
**Adoptive cell therapy**					
NCT05389514	Intermediate-size IND	I	AST KRAS^G12V^m	Chemo/immunotherapy	Available
NCT05933668	YK0901 TCR-T cell for KRAS G12V	I	AST		Not yet recruiting
NCT05438667	TCR-T cell therapy	I	PDAC		Recruiting
NCT06105021	Autologous CD8+/CD4+ TCR R cells	I/II	AST KRAS^G12V^m		Not yet recruiting
NCT04146298	Mutant KRAS G12V-specific TCR transduced T cell therapy	I/II	PDAC, KRAS^G12V^m	PD-1 inhibitor	Recruiting
NCT03745326	Peripheral blood lymphocytes with Murine KRASG12D TCR	I/II	PDAC/Gastric Ca/CRC	Cyclophosphamide, fludarabine, aldesleukin	Recruiting
NCT03190941	Peripheral blood lymphocytes transduced with Murine KRAS^G12V^ TCR in HLA-A*11:01	I/II	PDAC/Gastric Ca/CRC	Cyclophosphamide, fludarabine, aldesleukin	Recruiting
**Vaccines**					
NCT04117087	Mutant KRAS-targeted long peptide vaccine	I	Resected MMR-p CRC, PDAC	Ipilimumab, nivolumab	Recruiting
NCT03592888	Dendritic cell vaccine	I	Resectable PDAC		Active, not recruiting
NCT04853017-AMPLIFY-201	ELI-002 immunotherapy	I	Adjuvant KRAS/NRAS PDAC/ST with MRD		Active, not recruiting
NCT05013216	Mutant KRAS-targeted long peptide vaccine	I	High risk of PDAC		Recruiting
NCT05631899	EphA1-targeting CAR-dendritic cell vaccine	I	AST, EphA2 overexpression	Anti-PD1 antibody	Recruiting
NCT05846516-KISIMA-02	ATP1450/ATP152	I	KRASm PDAC	VSV-GP154, ezabenlimab	Recruiting
NCT05726864-AMPLIFY7P	ELI-002-7P	I/II	Adjuvant AST		Recruiting
NCT05638698-TESLA	TG01	II	Resected PDAC w/ctDNA+	QS-21, balstilimab	Recruiting
NCT06015724	Anti-CD38 antibody with KRAS vaccine	II	PDAC/NSCLC	Anti-PD1 antibody	Recruiting
**KRAS siRNAs**					
NCT03608631	Mesenchymal stromal cell-derived exosomes with KRAS G12D siRNA	I	Advanced PDAC KRAS ^G12D^m		Active, not recruiting

### Non-G12C allele-specific inhibitors

Given that G12D, G12V, and G12R represent over 90% of KRAS mutations in pancreatic cancer, an effective therapy for these cancers represents a significant unmet need.

There are multiple drugs targeting KRAS^G12D^ in development, with clinical trials for three compounds currently enrolling (Table 2). MRTX1133 is a selective, noncovalent G12D inhibitor which binds ionically to the aspartate 12 residue, inhibiting both the inactive and active states of KRAS^G12D^ (3). It is highly selective with a 700-fold affinity for KRAS^G12D^ versus wild-type (90) and has shown promising efficacy in mouse models with KRAS^G12D^ mutations, with >30% tumor regression in 8 of 11 PDAC models (90). The safety and efficacy profile of HRS-4642, a highly selective G12D inhibitor has been reported in a phase 1 trial of 18 patients, 15 of whom had NSCLC or CRC with an ORR of 33.3% (91). Furthermore, RMC-9805, another tricomplex inhibitor which targets the GTP-bound “ON” state of KRAS^G12D^ has shown promising preclinical results and studies have suggested synergy with immunotherapy (92). Other small molecules in development include TH-Z835, which forms a salt bridge with the aspartate 12 residue and binds to both inactive and active forms (93). This has shown encouraging efficacy in pancreatic mouse models however, the unintended side effect of weight loss suggests off-target effects.

### Pan-RAS inhibitors

In 2023, the first tri-complex pan-RAS inhibitor RMC-(94)6236 announced initial results from a phase I trial in PDAC and NSCLC (Table 2). Across multiple doses, the ORR in 46 heavily pre-treated PDAC patients was 20% with a DCR of 87%. The median time to response was 1.4 months (1.2–4.4 months) and median time on treatment was 3.3 months (0.2–10.9 months). Notably, in 12/13 PDAC patients circulating tumor DNA (ctDNA) analyses revealed >50% reduction in the mutated allele (94). Importantly, the drug was well tolerated with most treatment related adverse events (TRAEs), grade 1–2. RMC-6236 has demonstrated pre-clinical activity with immune checkpoint inhibitors and may overcome RAS oncogene switch resistance mutations (95). A related compound, RMC-7977 has shown broad-spectrum activity against both mutant and wild-type KRAS cell lines (96).

Additional pan-RAS inhibitors include the nanomolar compound BI-2852 which targets a second pocket at the switch I/II position and inhibits all three RAS isoforms (97). BI-2865 is a non-covalent inhibitor which binds to the GDP-bound state of a range of KRAS mutants while sparing HRAS and NRAS, suppressing growth in KRAS mutant tumors in mice (98). A further compound, RSC-1255 binds to vacuolar ATPase, inhibiting autophagy and macropinocytosis, and selectively destroying KRAS/BRAF mutant cells, in particular, KRAS^G13D^ and KRAS^G12V^ mutated cells (99). Clinical trials in progress of pan-RAS inhibitors are shown in Table 2.

### SOS1 and SHP2 inhibitors

The first small molecule SOS1 inhibitor to make it to the clinical setting is BI1701963, a derivative of BI-3406. It binds to the catalytic domain of SOS1 and prevents activation of KRAS without affecting SOS2-mediated signaling (100). In a phase I trial of 31 patients with KRAS mutated solid tumors, it was tolerable and showed stable disease in 7/31 patients at 18 months (101). Toxicity with these agents is a challenge and multiple trials have been terminated or stopped early by the sponsor (NCT04835714, NCT0462714) (102).

There is a plethora of SHP2 inhibitors currently at the clinical trial stage; many of these are first-in-human trials and yet to report (Table 2). A tri-complex SHP2 inhibitor, RMC-4630, demonstrated a disease control rate of 71% (5/7) and a reduction in tumor volume in 43% (3/7) in a small number of patients with KRAS^G12C^ mutated NSCLC (103). Subsequent analysis of ctDNA in this trial showed a decrease in KRAS^G12C^ variant allele frequency in 5/9 (59%) of patients who had detectable ctDNA at trial commencement, and this was associated with treatment response. Reduction in ctDNA was not seen in patients with KRAS^G12D^ or KRAS^G12V^ mutated tumors, therefore its potency as a single agent in pancreas cancer is uncertain (104). Clinical efficacy has been documented with TNO155, a pyrazine allosteric SHP2 inhibitor. In a phase I dose-finding study, 118 patients with advanced solid tumors were treated with TNO-155 with 20% experiencing stable disease (105). Preliminary results from the FLAGSHP-1 study, analyzing the SHP2 inhibitor ERAS-601 in advanced solid tumors were disappointing, with a partial response seen in 1/27 patients treated (106). These results suggest that SHP2 inhibitors may need to be used in combination to be effective. Notably, KRAS^G12R^ mutant and KRAS^Q61^ mutant tumors appear less sensitive to SOS1 inhibition and SHP2 inhibition (107).

## Combination strategies

### Combination with upstream inhibitors

Combination strategies are likely necessary to overcome the rapid acquisition of resistance and modest duration of response of direct KRAS inhibitors. Obtaining a sustained clinical response to direct KRAS inhibitors may require targeting GEFs by blocking receptor tyrosine kinases/SHP2/SOS1 or mitigating feedback activation of the pathway. Co-inhibition of KRAS^G12C^ and SHP2 has been shown to drive sustained RAS suppression and improve efficacy *in vitro* and *in vivo* (108). Jacobio et al. recently reported initial results from a phase I/IIa study of JAB-3312 in combination with the KRAS^G12C^ inhibitor glecirasib in patients with KRAS^G12C^ mutated solid tumors (109). In 28 patients with treatment-naïve NSCLC, ORR was 50% (14/28), with 100% DCR. A cohort of patients who had previous treatment with a KRAS^G12C^ inhibitor demonstrated an ORR of 14.3% (1/7). Tolerability will be critical here and the rate of Grade 3 and 4 TRAEs was notable at 36.7%.

### Combination with downstream inhibitors

Simultaneous blockade of downstream effectors to mitigate feedback activation and overcome resistance pathways is another potential combination strategy. There are ongoing clinical trials of direct KRAS^G12C^ inhibitors in combination with MEK and ERK inhibitors though preclinical evidence seems stronger for upstream inhibition (108). Moreover, inhibition of parallel pathway (e.g., PI3K/Akt/mTOR) downstream effectors may work more effectively. Preclinical evidence shows mTORC and KRAS^G12C^ inhibitors acted synergistically to increase cell death in mouse models (110). This will be further explored in clinical trials of sotorasib and the mTOR inhibitor, everolimus and adagrasib and the PIK3CA inhibitor, INCB099280 (Table 2).

### Combination with immunotherapy

Exploratory results from major immunotherapy trials have indicated that KRAS status impacts on response to checkpoint inhibition. In the KEYNOTE-042 and CA209-057 trials, patients with KRAS mutated tumors had superior responses to single-agent pembrolizumab and nivolumab, respectively, compared with KRAS wild-type (111, 112). However, this effect may have been dominated by KRAS^G12C^ mutations which respond more favorably to checkpoint inhibition than other KRAS mutations (113). Trials of checkpoint inhibition in PDAC have so far been unsuccessful, however, preclinical studies suggest that a rationale for combination with KRAS-directed therapies. KRAS^G12D^ inhibition with MRTX1133 alters the TME which may contribute to the anti-tumor effect with a reduction in myeloid cells, MDSCs and dendritic cells, an increase in M1/M2 macrophage ratio and stimulation of T cell infiltration (114). While the combination of sotorasib with immunotherapy (pembrolizumab or atezolizumab) resulted in a significant rate of hepatotoxicity (115), the addition of pembrolizumab to adagrasib in the KRYSTAL-07 trial appeared to increase response rates (ORR 49%) with an acceptable toxicity profile (116). Furthermore, SHP2 acts with the receptor PD-1 to have an immunomodulatory effect on T cells, therefore the combination of SHP2 with PD-1 inhibitor is promising and several clinical trials are ongoing (Table 2).

### Combination with other agents

Co-occurring inactivating mutations in the tumor suppressor CDKN2A result in the depletion of the cell cycle inhibitory protein p16^INK4a^. It was therefore hoped that CDK4/6 inhibitors, which act to chemically restore the function of p16^INK4a^, would be effective. The clinical response to CDK4/6 inhibitors in PDAC has been disappointing thus far, however, there is emerging evidence that a combination approach with MAPK inhibition may be efficacious. In preclinical PDAC mouse models, downstream MAPK inhibition with the MEK inhibitor, trametinib in combination with CKD4/6 inhibitor, palbociclib induced a senescent state, inhibiting PDAC growth (117). This senescent phenotype resulted in a modified TME with enhanced tumor vascularisation and increased levels of CD8+ cells, which may point to synergistic effects with chemoimmunotherapeutic strategies. Moreover, simultaneous co-inhibition of CDK2, a protein which acts with CDK4/6 to promote cell cycle progression, has been shown to have accentuated anti-tumor activity *in vivo* and *in vitro* (118–120), pointing to another potential combination strategy. In a similar vein, concurrent inhibition of CDK4/6 and ERK in PDAC organoid models resulted in apoptosis (119). There are clinical trials ongoing of CDK inhibitors in combination with MEK inhibitors [NCT05554367], ERK inhibitors [NCT03454035], direct G12C inhibitors, and SHP2 inhibitors (Table 2).

Additional agents currently at clinical trial stage in combination with KRAS inhibitors in PDAC include poly ADP ribose polymerase (PARP) inhibitors (olaparib), aurora kinase inhibitors (LY3295668), autophagy inhibitors (DCC-3116) and FAK inhibitor (IN10018) as well as traditional chemotherapy drugs.

## Novel strategies to target KRAS

### KRAS degraders

An innovative approach to target KRAS-driven cancers is to accelerate the destruction of mutant KRAS alleles. Proteolysis Targeting Chimeras (PROTACs) utilize the cell’s natural system for the destruction of damaged proteins (ubiquitin ligase) (121). These bivalent molecules form a complex with the mutant protein and E3 ubiquitin ligase. The E3 ligase then marks the mutant protein resulting in degradation (122). ASP3082, a PROTAC targeting KRAS^G12D^ has shown encouraging preclinical results with inhibition of growth in PDAC cancer cells. A phase I clinical trial is ongoing (123). It is likely that these agents will show a higher level of toxicity but may have a role in treating cancers driven by wild-type KRAS or amplified KRAS. PROTACs targeting SOS1 are also in development (124).

### siRNA

Small interfering RNAs (siRNAs) are short duplex RNA molecules that are introduced into target cells, inhibit the expression of specific messenger RNA, thereby causing gene silencing (125–128). At a cellular level, siRNA is specific, however delivery to target tissue can be challenging due to quick degradation, fast renal clearance and the dense stroma of PDAC (129, 130). One delivery approach is local intratumoral administration of siRNA. In a phase I/IIa study, Golan et al. enrolled 15 patients with locally advanced PDAC. They each had a biodegradable implant (Local Drug EluteR, LODER) containing siRNA targeting G12D inserted into their tumor and were treated synchronously with systemic chemotherapy (131). Response was seen in 2/12 analyzed by CT scans, with 10/12 exhibiting stable disease and median OS of 15.1 months (95% CI, 10.2–18.4). However, serious TRAEs were observed in 5/15 patients (131). Exosomes are an alternate delivery method. They are nano-sized extracellular vesicles that can be internalized by cells and used to deliver cargo (132). These exosomes have advantages over liposomes in terms of their efficiency and half-life due to CD47 on the surface (133). Enhanced macropinocytosis in KRAS mutant cells enables uptake of exosomes despite the dense stroma of pancreatic cancers (134). Kamerkar et al. successfully engineered exosomes to carry siRNA targeting KRAS^G12D^ and found that treatment inhibited cancer growth in advanced PDAC mouse models and increased overall survival (135).

### Adoptive cell therapy

In 2016, Tran et al. reported the first case of a KRAS^G12D^ mutated colorectal cancer treated with T cells recognizing neoantigen G12D-HLA-C*08:02. Regression was seen in all 7 metastases (136). More recently, the same group reported the case of a patient with KRAS^G12D^ mutated metastatic pancreatic cancer who was treated with autologous peripheral CD8+ and CD4+ T cells which had been engineered to express a T cell receptor (TCR) against the mutant KRAS^G12D^ (137). Treatment response was ongoing at 6 months. This is promising; however, the heterogeneity of KRAS-mutated pancreatic cancer may dampen the dramatic responses seen in other malignancies. The ability to select for neoantigens and the logistics of these strategies will pose global challenges.

## Cancer vaccination

Peptide vaccines are increasingly being developed to elicit an immune response in PDAC and may provide greatest benefit in the adjuvant setting. ELI-002 consists of amphiphile-modified KRAS mutant peptides (G12D and G12R) together with amphiphile-modified Toll-like receptor (TLR) 9 agonistic CPG-7909 DNA. The novel approach through its binding to endogenous albumin encourages transit from subcutaneous tissues into lymph glands rather than the bloodstream. In AMPLIFY-201, patients were treated who had ctDNA detected or elevation in serum biomarkers following completion of surgery/adjuvant therapy. Twenty-five patients were included and 84% demonstrated mutated KRAS-specific T cell responses. Reduction in ctDNA was seen in 77% with 33% showing clearance of ctDNA (138). AMPLIFY-7P will further investigate a related compound, ELI-002 7P in a phase I/II study.

TG01 is a vaccine containing seven synthetic RAS peptides that target KRAS. In phase I/II trial, 32 patients with resected stage I or II PDAC were treated with TG01/GM-CSF in combination with adjuvant gemcitabine. Over 90% developed an immune response as defined by a delayed-type hypersensitivity response and/or a positive T cell proliferation assay. Median OS was 33.1 months (95% CI 16.8, 45.8) (139). A phase II trial is underway (NCT 05638698). Another long peptide pooled mutant-KRAS vaccine is being investigated in a phase I trial in combination with nivolumab and ipilimumab in patients with resected MMR-proficient colorectal cancer and pancreatic cancer (NCT04117087).

The KISIMA vaccine platform includes 3 components: a cell-penetrating peptide for intracellular antigen delivery, a multi-antigenic cargo tailored to the tumor type and a TLR peptide agonist to enhance the immune response. A phase I trial is underway in which patients are treated with a ‘prime-boost’ vaccine, consisting of the protein vaccine described (ATP150 or ATP152) in combination with a viral vector VSV-GP154 and an immune checkpoint inhibitor (KISIMA-02). Initial testing will be done in locally advanced/metastatic KRAS^G12D^ or KRAS^G12V^ mutated PDAC, progressing to randomized parallel cohorts of resected PDAC and a control arm (NCT05846516). DC3/8 is a dendritic cell (DC) vaccine loaded with KRAS mutated peptide which is currently being investigated in resected PDAC patients. An accrual target of 29 patients will receive autologous DCs with mutant KRAS peptides according to the patient’s tumor mutation and HLA subtype (NCT03592888). The mRNA vaccine mRNA-5671/V941 is enclosed in a lipid nanoparticle which targets KRAS G12D, G12V, G13D, and G12C. The mRNA is absorbed by APCs and translated into peptides to be presented. This has elicited strong T-cell responses in mouse models (140, 141). A phase I in-human clinical trial of V941 alone or with pembrolizumab has completed accrual, and results are awaited (NCT03948763).

## Resistance mechanisms

Although KRAS mutations are prevalent across tumor types, it’s clear that there is heterogeneity with regard to responses. Understanding both innate and acquired resistance mechanisms to KRAS inhibitors is key to identifying optimal combination strategies and overcoming the modest duration of response we have seen with existing direct KRAS inhibitors. Adaptation to G12C inhibitors has been described as happening in a G12C-dependent or independent manner and can be rapid (142). Dependant adaptation occurs post treatment with G12C inhibitors, when some cancer cells are sequestered in an inactive state and newly formed G12C cancer cells are amplified and resume proliferation (143). Independent adaptation happens through a variety of mechanisms (89, 143). KRAS cells may acquire additional KRAS mutations on codons 12, 13, and 61. They may also acquire mutations on the switch II binding pocket, e.g., KRAS R68S, H95D/Q/R, Y96C/D (89, 143). These mutations may lead to noncovalent binding inhibition at the pocket site. Interestingly, some pocket site mutations which appeared after adagrasib treatment, conferred marked resistance to adagrasib however remained sensitive to sotorasib (143). Therefore, resistance to some inactive-state inhibitors can be overcome by a functionally distinct KRAS^G12C^ inhibitor (89). In addition, the tricomplex inhibitors may overcome resistance from mutations in the switch II pocket.

Secondary bypass alterations in the RAS/MAPK/PI3K signaling pathway in patients who have progressed on sotorasib/adagrasib have also been documented (143, 144). These included treatment-emergent amplifications, mutations or fusions of genes encoding receptor tyrosine kinases MET, EGFR, RET, ALK, and FGFR3 leading to investigation of combination strategies. However, the specific receptor tyrosine kinase driving the rebound in signaling can vary widely among tumors suggesting it might be advantageous to look at downstream blockade. Other bypass mechanisms include mutations or fusions of downstream effector kinases: BRAF, CRAF, and MEK1; loss of function mutations in NF1 and PTEN; NRAS and PI3K mutations. Cell state transitions are an additional mechanism of resistance and in NSCLC cell lines epithelial to mesenchymal transition was responsible for both intrinsic and acquired resistance (145). The contribution of the TME and stromal compartment in resistance is yet to be established.

The diversity of resistance mechanisms that have emerged in response to KRAS G12C inhibition highlights the challenges in targeting RAS. As data on pan-RAS inhibitors emerge, these will no doubt expand, underscoring the need for rapid adaptive combination approaches in the trial setting.

## Future directions/conclusion

Targeting the RAS pathway remains the biggest challenge in precision oncology. The future in PDAC will likely see selection of first line combinations based on KRAS allelic status.

Vaccine strategies herald huge promise, especially in early-stage disease. The modest duration of response and emergence of resistance mechanisms to the KRAS^G12C^ inhibitors points to challenges that we are likely to see across the direct KRAS inhibitors. Overcoming resistance and defining optimal combination strategies is likely to be crucial to improving patient outcomes. This requires an in-depth knowledge of not just the mutational profile but of metabolic needs and distinct TMEs of each patient. The plethora of new agents under investigation has the potential to revolutionize the treatment paradigm for patients with KRAS-mutated pancreatic cancer.

## Author contributions

AL: Writing – review & editing, Writing – original draft. MO’R: Writing – review & editing, Writing – original draft. RM: Writing – review & editing, Writing – original draft. GO’K: Writing – review & editing, Writing – original draft.

## Glossary

ACT: Adoptive cell therapy
AST: Advanced solid tumors
CAF: Cancer-associated fibroblasts
CtDNA: Circulating tumor deoxyribonucleic acid
CRC: Colorectal cancer
DC: Dendritic cell
DCR: Disease control rate
DOR: Duration of response
FOLFIRI: 5-fluorouracil, leucovorin, irinotecan
FOLFIRINOX: 5-fluorouracil, leucovorin, irinotecan, oxaliplatin
EGFR: Epidermal growth factor receptor
ERK: Extracellular signal-regulated kinase
GAP: Guanosine triphosphate activating protein
GDP: Guanine diphosphate
GEF: Guanine exchange factors
GM-CSF: Granulocyte macrophage colony stimulating factor
GRB2: Growth factor receptor-bound protein 2
GTP: Guanine triphosphate
HRAS: Harvey rat sarcoma
KRAS: Kirsten rat sarcoma
KC: Kras^LSL-G12D/+^ Pdx-Cre
KPC: Kras ^LSL-G12D/+Trp53LSL-R172H/+^ Pdx-Cre
LODER: Local drug EluteR
IPMN: Intraductal papillary mucinous neoplasm
MAPK: Mitogen activating protein kinase
MDSC: Myeloid derived suppressor cells
MMR: Mismatch repair
MMR-p: Mismatch repair proficient
MOS: Median overall survival
MPFS: Median progression free survival
MRD: Minimal residual disease
NSCLC: Non-small cell lung cancer
NF-1: Neurofibrin-1
NR: Not reported
NRAS: Neuroblastoma rat sarcoma
ORR: Objective response rate
OS: Overall survival
PanIN: Pancreatic intraepithelial neoplasia
PARP: Poly ADP ribose polymerase
PDAC: Pancreatic ductal adenocarcinoma
PFS: Progression free survival
PI3K: Phosphoinositide 3-kinase
RAF: Rapidly accelerating fibrosarcoma
RALGDS: Ral guanine nucleotide dissociation stimulator
RASGRF2: RAS protein-specific guanine nucleotide releasing factor 2
RBD: RAS binding domain
SCLC: Small cell lung cancer
SHP2: Src homology-2-domain-containing protein tyrosine phosphatase-2
SiRNA: Small interfering ribonucleic acid
SOS: Son of sevenless
TAM: Tumour associated macrophage
TCA: Tricarboxylic acid
TCF: T cell factor
TCR: T cell receptor
TME: Tumor microenvironment
TLR: Toll-like receptor
TRAE: Treatment related adverse event
Treg: Regulatory T cell
PROTAC: Proteolysis targeting chimera
